# Dietary intakes of citrus fruit and risk of gastric cancer incidence: an adaptive meta-analysis of cohort studies

**DOI:** 10.4178/epih.e2016034

**Published:** 2016-07-25

**Authors:** Jong-Myon Bae, Eun Hee Kim

**Affiliations:** Department of Preventive Medicine, Jeju National University School of Medicine, Jeju, Korea

**Keywords:** Gastric neoplasms, Risk factors, Citrus fruit, Meta-analysis

## Abstract

**OBJECTIVES:**

In the context of supplementary antioxidants having no anticancer effect, it is important to update the meta-analysis to evaluate whether there is an association between intake of citrus fruit and gastric cancer risk.

**METHODS:**

The list of articles to be searched was established using citation discovery tools provided by PubMed and Scopus. The effect size of each article to be used in meta-analysis was calculated using the interval-collapse method. Summary effect size (sES) and 95% confidence intervals (CI) were obtained by conducting this meta-analysis. Random effect dose–response meta-regression (DRMR) was performed to investigate the dose–response relationship.

**RESULTS:**

A total of five cohort studies were selected. The result was 13% reduction of gastric cancer according to the intake of citrus fruit (sES, 0.87; 95% CI, 0.76 to 0.99; I-squared=69.6%). In subgroup analysis, it was found that the intake of citrus fruit inhibited cardia gastric cancer (CGC) (sES, 0.67; 95% CI, 0.55 to 0.81; I-squared=46.1%) and as a result of DRMR, 100 g of citrus fruit intake per day inhibits CGC by 40% (relative risk, 0.60; 95% CI, 0.44 to 0.83).

**CONCLUSIONS:**

It is suggested that the intake of citrus fruit inhibits the development of CGC. This conclusion can be used as a primary prevention measure in the future when the incidence of CGC may be on the rise.

## INTRODUCTION

As one of the main types of primary cancers, gastric cancer is the fourth and fifth most common cancer worldwide in men and women, respectively, and has the highest incidence rate in Far East Asia, which includes Korea [1]. The observance of such geographical characteristics has been attributed to chronic *Helicobacter pylori* (Hp) infection [2-4]. However, the fact that less than 0.5% of patients with Hp infection acquire stomach cancer suggests that other risks or protective factors may be involved in contributing to this profile of geographical characteristics [5-7].

In 2008, Bae et al. [8] published a systematic review (SR) of epidemiologic studies that had been published until April 2007; the SR investigated the relationship between citrus fruit intake and gastric cancer risk. This meta-analysis, which reviewed a total of 14 analytical epidemiological studies, showed that dietary intake of citrus fruit reduced gastric cancer risk by 28% (summary effect size [sES], 0.72; 95% confidence intervals [CI], 0.64 to 0.81). However, results of two cohort studies included in the meta-analysis lacked statistical significance (sES, 0.87; 95 CI, 0.67 to 1.13). It was concluded that since only a small number of papers were published on this topic, and case-control studies tend to contain more epidemiological errors than cohort studies, additional studies would be needed in the future [9,10].

On the one hand, Fang et al. [11] published SR results in 2015 on cohort studies that had been published until June 2015 that identified dietary factors associated with gastric cancer risk. The meta-analysis results on eight cohort studies [12-19] with regard to citrus fruit intake were marginally significant with sES 0.90 (95% CI, 0.82 to 1.00). However, among these eight selected cohorts, Botterweck et al. [12] and Steevens et al. [17] investigated the same cohort called as the Netherlands Cohort Study, and likewise, González et al. [13] and Gonzalez et al. [16] investigated the same cohort named as the European Prospective Investigation into Cancer and Nutrition Study. That is to say, Fang et al. [11] conducted a meta-analysis without taking into account the redundancy of cohort sources. In order to obtain valid results, cohort studies with short follow-up periods among cohort studies with the same participants [12,13] should be excluded from the analysis. Moreover, analyzing cohort studies that look at cancer mortality alongside those that look at cancer incidence, as in the case of McCullough et al. [14] and Jansen et al. [15], poses a problem to epidemiological inference [20]. For these reasons, Fang et al. [11] needs to re-evaluate and re-meta-analyze the cohort studies selected for the meta-analysis. Namely, there is a need to perform an adaptive meta-analysis.

Therefore, this study aims to perform an adaptive meta-analysis on cohort studies published until December 2015 in order to overcome the shortcomings of the two previous SRs [8,11] and improve validity.

## MATERIALS AND METHODS

### Related article search and selection

Cohort studies that investigated the relationship between dietary intake of citrus fruit and gastric cancer risk were selected for this study. Related articles were selected through a three-step process involving searching in databases, examining titles and abstracts, and reviewing literature content, as suggested by preferred reporting items for SRs and meta-analyses [21].

The data searching was done manually rather than electronically. This is because two SRs [8,11] had been previously published, and accordingly, it would be more efficient to do an adaptive meta-analysis that updates the past literature [22-25]. In Bae et al. [8] and Fang et al. [11], for eight articles selected for meta-analysis, lists of articles to be searched were created by using the citation discovery tools (CDT) that served ‘Cited’ ‘Similar’ and ‘Related’ options, provided by PubMed (http://www.ncbi.nlm.nih.gov/pubmed) and Scopus (www.scopus.com). The final publication date was set to December 31, 2015. In addition, manual searching was done to check if a SR had already been published on the hypothesis in question, and more lists were created. Lists made manually and through the CDT were combined, and duplicated lists were eliminated.

For the screening stage, studies were excluded if they fell into the following criteria based on their titles and abstracts: (1) laboratory experiments, (2) expert/SRs, and (3) descriptive studies. Studies that passed the screening process then moved on to the third stage for eligibility assessment. Copies of each study were collected and evaluated, and cohort studies that met the following criteria were sequentially excluded: (4) analytical studies that did not provide information necessary for the meta-analysis, (5) case-control studies, (6) gastric cancer mortality-based cohort studies, and (7) cohort studies that were duplicated as a result of extending a follow-up period. The remaining studies after applying the seven exclusion criteria were selected as the final articles to be used in the meta-analysis.

### Statistical analysis

For each study, we looked at the cohort data source, the average length of follow-up period, the number of participants in the cohort as well as the number of cases who developed cancer, the method of investigating the quantity and unit of dietary citrus fruit intake, the adjusted relative risk (aRR) obtained after adjusting for confounders for each distribution of dietary intake and the corresponding 95% CIs, and whether any of the adjusted confounding factors controlled the total energy intake and history of Hp infection. In the cases where an aRR was reported separately based on sex (male, female) and anatomical region (cardia, non-cardia), we considered the aRRs as separate datasets instead of combining them into one.

For aRR of each dataset to be used in the meta-analysis, the ‘interval collapsing’ method (ICM) was used instead of the ‘highest vs. lowest intake’ method (HLM). This is because ICM utilizes a greater amount of information than HLM and therefore improves statistical accuracy [26]. In an ICM, effect sizes are suggested for each level of intake of citrus fruits within a dataset of a study, and an effect size obtained from a meta-analysis of a random effect model (REM) and the corresponding 95% CI are set as the effect size of the study. A REM meta-analysis was performed again on the effect size of each study to calculate an sES and the corresponding 95% CI. The meta-analysis was considered to contain heterogeneity if I-squared value (%) was greater than or equal to 50%. To detect any publication bias, a funnel plot and Egger’s test for small-study effects were performed [27,28]. Additionally, a subgroup analysis was performed for each sex and stomach region.

In addition, in order to study the dose–response relationship for different quantities of citrus fruit intake, random effects dose– response meta-regression (RE-DRMR) was performed [29]. For dose determination, a median value within each interval was used, and the lower bound value was set as zero if the lowest intake interval was open. If the highest intake interval was open, a median value of the adjacent interval was added to the low boundary of the interval [30,31]. The unit of citrus fruit intake was set as grams/day (g/d). Level of statistical significance was set at 5%, and the Stata version 14.0 (StataCorp, College Station, TX, USA) statistical program was used (www.stata.com).

## RESULTS

**Figure 1.**
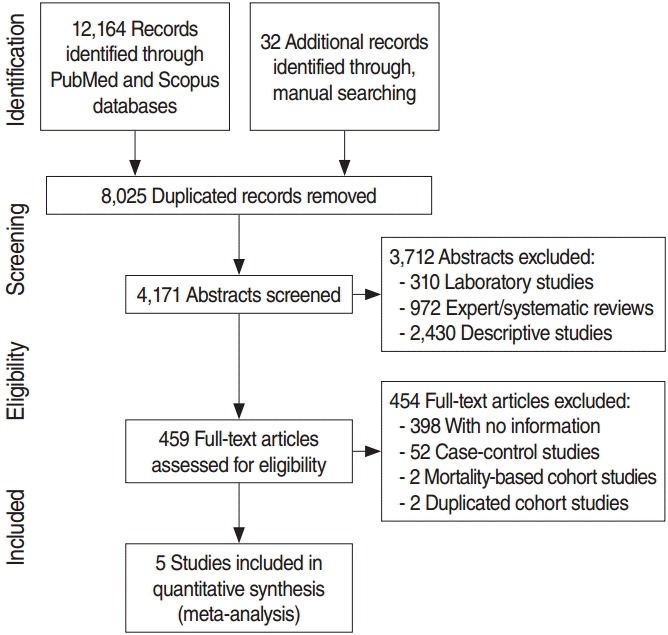
Flow chart of article selection.

Figure 1 shows a flow chart illustrating a series of steps involved in the final selection of articles to be used in the analysis including the searching and evaluating processes. After applying the CDT on the two databases (PubMed and Scopus) based on the eight articles selected in the meta-analysis in Fang et al. [11], we obtained 12,164 records. After combining these records with 32 other records identified through manual searching, we eliminated 8,025 duplicated records. Among the 4,171 remaining records, 3,712 were eliminated based on abstract contents. Copies of the remaining 459 abstracts were obtained and their contents screened, leading to elimination of 454 articles. Five cohort studies were finally selected for the meta-analysis [16-19,32]. A total of 4,166 articles were excluded according to the exclusion criteria: (1) 310 laboratory studies, (2) 972 expert/SRs, (3) 2,430 descriptive studies, (4) 398 analytical studies that do not provide information necessary for the meta-analysis, (5) 52 case-control studies, (6) 2 gastric cancer mortality-based cohort studies [14,15], and (7) 2 cohort studies that were duplicated as a result of extending the follow-up period [12,13].

Table 1 summarizes the characteristics of the five cohorts that were finally selected. Geographical regions included the US, Japan, China, the Netherlands, and Europe. The follow-up period length was 4.5 years at the minimum, and 11 years at the maximum, and the quantity of citrus fruit intake was measured by food frequency questionnaire (FFQ). aRR calculation was adjusted for smoking in all five articles, and it was also adjusted for total energy intake in four of the articles (Steevens et al. [17] not included). It was not adjusted for Hp infections in all of the articles.

**Figure 2.**
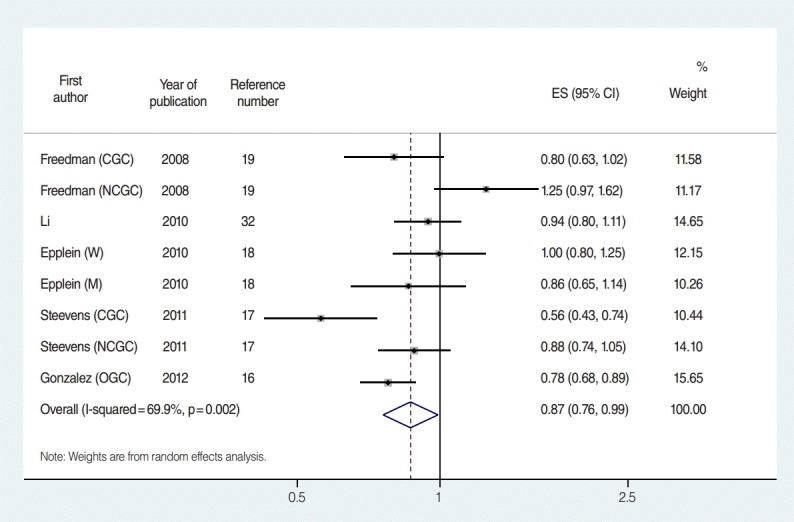
The forest plot of effect size (ES) and its 95% confidence intervals (CI) using a random effect model in eight datasets from five cohort studies. M, men; W, women; CGC, cardia gastric cancer; NCGC, non-cardia gastric cancer.

**Figure 3.**
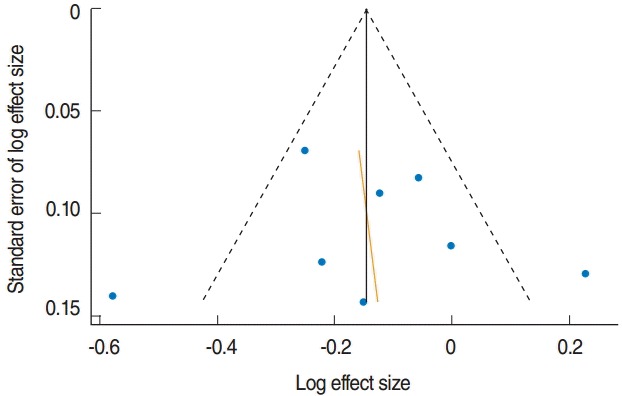
Funnel plot with pseudo 95% confidence limits in eight datasets from five cohort studies.

Figure 2 is a forest plot showing effect size with 95% CI obtained by applying the ICM on aRRs and their corresponding 95% CIs in eight databases from five cohorts based on sex and anatomical region. Gonzalez et al. [16] not only calculated aRRs for the anatomical regions, but also reported an overall aRR pertinent to the total cohort population, so that the relative risks (RRs) for overall gastric cancer were used in the meta-analysis for estimating overall effect. The sES of the eight datasets was 0.87 (95% CI, 0.76 to 0.99; I-squared=69.9%), and citrus fruit intake inhibited gastric cancer development with statistical significance. An Egger’s test was performed because moderate heterogeneity was detected. Small study effects were negligible (coefficient of bias=0.43; p-value=0.89), and bilateral symmetry could be observed in the funnel plot (Figure 3).

In the subgroup analysis of each anatomical region, it was found that non-cardia gastric cancer had no statistical significance in relation to citrus fruit intake (sES, 0.95; 95% CI, 0.78 to 1.15; I-squared=77.5%). However, citrus fruit intake had inhibitory effects on the development of cardia gastric cancer (CGC), and also had a statistically significant relationship with CGC risk (sES , 0.67; 95% CI, 0.55 to 0.81; I-squared=46.1%) (Table 2). Of the five cohort studies, Steevens et al. [17] provided information regarding CGC that could be used in a RE-DRMR. Table 3 shows the RE-DRMR results. There was statistical significance in the relationship between citrus fruit intake and CGC risk (p-value=0.002), and it was found that an intake of 100 g of citrus fruit per day reduced CGC risk by 40% (RR, 0.603; 95% CI, 0.439 to 0.827).

## DISCUSSION

To summarize the results, dietary intake of citrus fruit reduced gastric cancer risk by 13%, and had an inhibitory effect on CGC in particular. Since the subgroup analysis and RE-DRMR analysis showed that citrus fruit intake has no statistical significance with respect to NGC, it can be inferred that citrus fruit intake inhibits the development of CGC. With antioxidant supplement found to have no anticancer effects [33-35], the result of this study becomes a positive evidence for gastric cancer prevention.

The present study differs from the SR done by Fang et al. [11] in terms of the two methodologies used. First, whereas Fang et al. [11] used electronic searching during the data searching process, our study used the CDT strategy [26]. This strategy was developed while taking into account the fact that studies that share the same hypothesis cite articles that have already been selected in existing SRs, and are also similar in their contents. Therefore, we were able to additionally obtain a study [32] that should have been selected in Fang et al. [11], and also exclude two duplicated cohort studies [12,13] that used the same cohort source. In other words, an adaptive meta-analysis method using the CDT had a lower omission rate compared to electronic searching, and allowed for a more valid selection of studies [22,23]. Secondly, our study differs in that it uses the ICM approach rather than the HLM approach to extract datasets from the selected studies to be used in the meta-analysis [26]. As a result, the directionality of sES in our study was closer towards “against null” than Fang et al. (0.90 vs. 0.87) [11]. Our study had a wider CI (0.82 to 1.00 vs. 0.76 to 0.99), but this could not be included in the comparison because our study excluded two cohort studies [14,15] that were mortality-based. After adding in these two cohort studies, the sES using ICM was on par with that in Fang et al. [11] at 0.90, and the 95% CI narrowed to 0.83 to 0.97 (Table 2). In other words, by using the ICM approach, we were able to further increase statistical power and obtain more accurate results [26].

This study has some limitations. First, none of the five selected cohort studies was adjusted for Hp infection. This may be due to the difficulty in performing Hp tests on all of the study participants during cohort establishment, with the importance of Hp infections being acknowledged only recently [10,36]. However, it is possible to obtain information regarding Hp infection and adjust for it through nested case-control studies (NCCS). A SR of 12 studies that utilized this NCCS method suggested that Hp infection was not associated with CGC risk (sES, 0.99; 95% CI, 0.72 to 1.35) [37]. If we accept this conclusion, it would be valid to say that citrus fruit intake has inhibitory effects on CGC even if the five cohort studies selected in our study were not adjusted for Hp infections. On the other hand, Hansen et al. [38] concluded that Hp infection-induced gastric atrophy is a risk factor of CGC, while Hp infection is not a direct risk factor of CGC. It is necessary to perform additional SRs on studies that were performed based on a NCCS design. Secondly, there were still not enough cohort studies selected to draw any results regarding CGC in different anatomical regions. Two studies were selected from the studies that were published until April 2007 [8], and five were selected from the studies that were published until December 2015 [11]. Although Bae et al. [8] could not establish any statistical significance in the relationship between citrus fruit intake and gastric cancer development in the meta-analysis, our study, with the addition of three more articles, was able to gain statistical significance. However, only three of the five selected cohort studies were used when we looked at gastric cancer risk for each anatomical region [16,17, 19]. Since no statistical significance was observed for NGC in our meta-analysis, it would be necessary to extend the cohort follow-up period and do a pooled analysis. Third, only one study provided information that could be used in the RE-DRMR. However, it was found that 100 g/d of citrus fruit intake reduced risk of gastric cancer incidence by 40%. On the other hand, Vingeliene et al. [39] reported that the same amount of citrus fruit intake could not lower mortality risk of gastric cancer (RR, 0.95; 95% CI, 0.85 to 1.05). More studies must be done with additional information regarding cohort studies.

## CONCLUSION

To summarize the results, dietary intake of citrus fruit inhibits the development of gastric cancer, especially CGC. With the growing trend in the incidence of CGC and the decreasing trend in the incidence of NGC [20], this finding may be used as part of a dietary improvement strategy for gastric cancer prevention.

## Figures and Tables

**Table 1.** The selected cohort studies for gastric cancer incidence

First author (year of publication) [reference number]	Cohort population	Size of cohort & Incident cases (year of follow-up)	Measure of intake (units)	Citrus fruits	Quantity of intake	Adjusted RR	95% CI	p for trend	Adjusted for total energy intake	Adjusted for *Helicobacter pylori* infection
Freedman (2008) [19]	NIH-AAPR Diet and Health Study	490,802 & 394 (4.5)	FFQ (daily serving /1,000 K calories)	Oranges, tangerines, tangelos, grapefruits	0.08	1.00	Cardia	-	Yes	No
					0.46	0.73	0.52, 1.03			
					1.12	0.88	0.62,1.23			
					0.08	1.00	Non-cardia	-		
					0.46	1.15	0.80, 1.67			
					1.12	1.36	0.96, 1.94			
Li (2010) [32]	Ohsaki Cohort Study	42,470 & 313 (9)	FFQ (times)	Citrus	≤2	1.00	-	0.63	Yes	No
					3-4	0.95	0.76, 1.18			
					Daily	0.94	0.74,1.20			
Epplein (2010) Shanghai [18]	Shanghai Women’s and Men’s Health Studies	132,311 & 338 (13)	FFQ (g/d)	Tangerines, organs, grapefruit	≤6.1	1.00	Women	0.86	Yes	No
					>6.1-17.7	1.00	0.68, 1.46			
					>17.7-31.9	1.05	0.71,1.53			
					> 31.9	0.94	0.62, 1.42			
					≤1.6	1.00	Men	0.34		
					> 1.6-≤6.3	0.84	0.52, 1.36			
					>6.3-≤18.0	1.02	0.65, 1.62			
					≤18.0	0.70	0.41,1.18			
Steevens (2011) [17]	The Netherlands Cohort Study on diet and cancer	4,035 & 616 (16.3)	FFQ (g/d)	Lemon (juice), grapefruit (juice), mandarins, orange (juice)	0	1.00	Cardia	0.003	No	No
					8	0.76	0.47, 1.22			
					33	0.54	0.32, 0.92			
					77	0.55	0.32, 0.94			
					156	0.38	0.21,0.69			
					0	1.00	Non-cardia	0.46		
					8	0.86	0.61, 1.21			
					33	0.89	0.62, 1.27			
					77	0.99	0.70,1.40			
					156	0.80	0.56, 1.15			
Gonzalez (2012) [16]	EPIC-EUR-GAST	477,312 & 683 (11)	FFQ	Citrus	Q1	1.00	Overall	0.07	Yes	No
					Q2	0.78	0.62, 0.99			
					Q3	0.84	0.67, 1.07			
					Q4	0.63	0.49, 0.82			
					Q5	0.87	0.68, 1.12			
					Q1	1.00	Cardia	0.01		
					Q2	0.73	0.49, 1.08			
					Q3	0.73	0.48, 1.11			
					Q4	0.54	0.34, 0.85			
					Q5	0.61	0.38, 1.00			
					Q1	1.00	Non-cardia	0.46		
					Q2	0.93	0.64, 1.34			
					Q3	1.07	0.75,1.53			
					Q4	0.79	0.54, 1.16			
					Q5	1.25	0.86, 1.80			

RR, relative risk; CI, confidence interval; NIH-AARP, National Institutes of Health-American Association of Retired Person; EPIC-EURGAST, European Prospective Investigation into Cancer and Nutrition; FFQ, food frequency questionnaire.

**Table 2.** Subgroup analysis by anatomical location of stomach cancer

Subgroup	Reference number	sES	95% CI	I-squared (%)
Incidence	16-19, 32	0.87	0.76, 0.99	61.3
Non-cardia	16, 17, 19	0.95	0.78, 1.15	77.5
Cardia	16, 17, 19	0.67	0.55, 0.81	46.1

sES, summary effective size; CI, confidence interval.

**Table 3.** Relative risks (RRs)^1^ of dose–response meta-regression using RRs of cardia gastric cancer in Steevens et al. [17]

Intake of citrus fruits (g/d)	RR	95% confidence interval
1	0.995	0.992, 0.998
10	0.951	0.921, 0.981
20	0.904	0.848, 0.963
25	0.881	0.814, 0.924
50	0.776	0.663, 0.910
75	0.684	0.540, 0.868
100	0.603	0.439, 0.827

1 p-value=0.002.
