# Supplementary material for: Dietary intakes of citrus fruit and risk of gastric cancer incidence: an adaptive meta-analysis of cohort studies
Source: Epidemiol Health. 2016 Jul 25;38:e2016034. doi: 10.4178/epih.e2016034 (PMC5037356; doi:10.4178/epih.e2016034)
Supplement: Supplementary file 1 [file epih-38-e2016034-supplementary.pdf]

<Systematic Review>

Dietary Intakes of Citrus Fruit and Risk of Gastric cancer

# : An Adaptive Meta-analysis of Cohort studies

감귤섭취와 위암발생 위험: 코호트연구에 대한 갱신 메타분석

Jong-Myon Bae, Eun Hee Kim

배종면, 김은희

Department of Preventive Medicine, Jeju National University School of Medicine

제주대학교 의전원 예방의학교실

Corresponding authors: Jong-Myon Bae

102 Jejudaehakno, Jeju-si, Jeju-do, 63241, Republic of Korea

jmbae@jejunu.ac.kr

Phone: +82-64-755-5567

Fax: +82-64-725-2593

Running Title: Citrus Fruit & Gastric Cancer

Abstract

Background: Under the situation that antioxidant supplementary has no anticancer effect, updating meta-analysis to evaluate association between intake of citrus fruit and gastric cancer risk.

Methods: The list of articles to be searched was established using citation discovery tools provided by PubMed and Scopus. The effect size of each article to be used in meta-analysis was calculated using interval-collapse method. Summary effect size (sES) and 95% confidence intervals (CI) were obtained by conducting meta-analysis. Random effect dose-response meta-regression (DRMR) was performed to investigate the dose-response relationship.

Results: A total of 5 cohort studies were selected. The result was 13% reduction of gastric cancer according to the intake of citrus fruit (sES=0.87, 95% CI: 0.76-0.99, I-squared = 69.6%). In sub-group analysis, it was found that the intake of citrus fruit inhibited cardia gastric cancer (CGC) (sES= 0.67, 95% CI: 0.55-0.81, I-squared=46.1%) and as a result of DRMR, 100gram citrus fruit intake per day inhibits CGC by 40% (RR=0.60, 95% CI: 0.44-0.83).

Conclusions: It is suggested that the intake of citrus fruit inhibits the development of CGC. This conclusion can be used as a primary prevention measures in the future under the situation that the incidence of CGC tends to increase currently.

Keyword: Gastric neoplasms, Risk factor, Citrus fruit, Meta-analysis

Introduction

전 세계적으로 위암은 남자에서 4번째, 여자에서 5번째로 높은 발생률을 보이는 주요 원발암 중의 하나로, 한국을 포함한 극동 아시아에서 가장 높은 발생률을 보이고 있다 [1]. 이처럼 지역적 특성을 보이는 이유 중의 하나로 *Helicobacter pylori* (Hp) 만성 감염을 지적하고 있다 [2-4]. 그러나 Hp 감염자 중 0.5% 미만에서 위암이 발생한다는 사실은 또 다른 위험 혹은 보호 요인이 관여한다고 볼 수 있다 [5-7].

Bae et al. [8]는 2007년 4월까지 발표된 감귤섭취와 위암발생간의 관련성을 규명한 역학연구들에 대한 체계적 고찰 (systematic review, SR) 연구 결과를 2008년도에 발표하였다. 총 14개의 분석역학연구를 대상으로 한 메타분석결과 감귤 섭취는 위암발생을 28% 감소시키는 것으로 제시하였다 (summary effect size, sES = 0.72; 95% confidence intervals, CI: 0.64-0.81). 그러나 이중 2개의 코호트 연구결과의 메타분석결과는 통계적 유의성을 보이지 않았다 (sES = 0.87; 95 CI: 0.67-1.13). 논문 수가 적고 코호트 연구보다 환자-대조군 연구가 더 역학적 오류가 많다는 점에서 향후 추가적인 연구가 필요하다고 결론지었다 [9,10].

한편, Fang et al. [11]은 2015년 6월까지 발표된 코호트 연구로 위암발생에 있어 식이요인을 알아본 것들을 SR한 결과를 2015년에 발표하였다. 이중 감귤에 있어서 8개의 코호트 [12-19]에 대한 메타분석 결과는 sES 0.90 (95% CI: 0.82-1.00) 으로 marginally significant 하였다. 그런데, 선정한 8개 코호트 중, Botterweck et al. [12] 와 Steevens et al.

[17] 은 the Netherlands Cohort Study란 동일 코호트에서 연구한 논문이며, Gonzalez et al. [13]과 Gonzalez et al. [16]은 the European Prospective Investigation into Cancer and Nutrition Study 란 동일 코호트에서 연구한 논문이다. 즉 코호트 자료원 (cohort source)의 중복을 고려하지 않고 메타분석을 한 것이다. 타당한 결과를 얻기 위해서는 이들 중 추적기간이 더 짧은 코호트 연구들은 제외해야 한다 [12,13]. 또한 McCullough et al. [14] 와 Jansen et al. [15] 는 위암의 사망 (mortality)을 결과로 본 코호트 연구로 발생 (Incidence)를 결과로 본 연구와 묶어서 분석하는 것은 역학적 추론 (epidemiological inference)에 문제가 된다 [20]. 이처럼 Fang et al. [11] 이 메타분석을 위해 선정한 논문들에 대하여 재검토 및 재분석을 할 필요가 있다. 다시 말해서 메타역학연구를 위하여 갱신용 메타분석 (adaptive meta-analysis)를 수행할 필요가 있다.

따라서 감귤섭취에 따른 위암발생 위험을 규명하려는 기존의 두 SR [8,11]의 한계를 극복하고 보다 타당한 결과를 얻어내기 위하여, 2015년 12월 이전까지 발표된 코호트 연구들을 대상으로 갱신용 메타분석을 시행하는 것이 본 연구의 목적이다.

Subjects & Methods

가. 관련한 논문 검색 및 선정

본 연구의 선정기준은 감귤섭취 수준에 따른 위암발생의 관련성을 규명한 코호트 연구 논문이다. 관련 논문을 선정하기 위하여 preferred reporting items for systematic reviews and meta-analyses 의 제안 따라 자료원에서의 검색, 제목과 초록을 이용한 검토, 문헌내용 고찰의 3 단계로 나누어 진행하였다 [21].

1단계 자료 검색과정은 전자검색보다는 손 검색 방법을 적용하였다. 이미 두 개의 체계적 고찰 [8,11]이 발표되었기에, 이의 updating 성격을 가진 adaptive meta-analysis를 하는 것이 보다 더 효율적이기 때문이다 [22-25]. 이에 Bae et al. [8] 와 Fang et al. [11] 에서 메타분석을 위해 선정한 총 8개 논문에 대하여 PubMed (http://www.ncbi.nlm.nih.gov/pubmed) 와 Scopus (www.scopus.com)이 제공하는 'Cited' 'Similar' 'Related' 기능인 Citation discovery tools (CDT)를 적용하여 검색 목록을 작성하였다. 이때 최종 출판일을 2015년 12월 31일로 한정하였다. 또한, 선정한 가설에 따라 체계적 고찰이 이미 발표된 것이 있는가를 알아보기 위해 손 검색을 하면서 목록을 추가로 만들었다. 이상의 CDT와 손 검색으로 만들어진 목록들을 합쳐 중복된 것을 우선 제거하였다.

2단계 스크리닝 작업을 위하여 앞서 정리된 목록에서 제목과 초록에 근거하여 다음의 제외 기준에 맞는 것을 제외시켰다. (1) 실험실 연구 (2) 전문가 종설 혹은 체계적 고찰 연구 (3) 기술역학연구. 2단계를 거치면서 제외한 후 남은 논문들에 있어 3단계 논문평가 작업을 수행하였다. 이를 위해 각각의 복사본을 확보하여 그 내용을 평가하면서 다음의 제외 기준에 맞는 것을 순차적으로 제외시켰다. (4) 분석역학연구이지만 메타분석에 필요한 정보를 제공하지 않은 연구 (5) 환자-대조군 연구 (6) 위암 사망에 관한 코호트 연구 (7) 추적기간을 연장한 코호트 추적 결과가 발표되어 중복된 코호트 연구. 이상의 7가지 제외 기준들을 적용하고도 남게 된 코호트 연구 논문들을 메타분석을 위한 최종 논문으로 선정하였다.

나. 통계분석

각 논문별로 코호트 자료원, 평균 추적 기간, 코호트 입적 대상자 및 위암 발생자수, 감귤섭취 수준 분석법 및 단위, 감귤섭취 분포별로 교란변수들을 보정한 adjusted relative risk (aRR)와 이의 95% confidence intervals (CIs), 그리고 보정한 교란변수 중 총에너지 섭취와 Hp 감염력을 통제하였는가 여부를 추출하였다. 성별 (남,여)과 부위 (cardia, non-cardia)로 나누어 aRR을 제시한 경우 이를 합치기보다는 각각의 dataset 로 간주하고 정리하였다.

메타분석에 사용할 각 dataset 의 aRR은 'highest versus lowest intake' method (HLM) 보다는 'interval collapsing' method (ICM)을 적용하여 선정하였다. 그 이유는 ICM 이 HLM 보다 더 많은 정보를 활용하게 되어 통계적 정확성이 향상되기 때문이다 [26]. ICM 적용은 한 dataset논문 내에서 BCX 수준별로 제시한 효과크기들을 random effect model (REM)의 메타분석을 수행하여 얻어낸 effect size (ES)와 이의 95% CI을 해당 논문의 효과크기로 삼는 것이다. ICM 법으로 구한 각 논문의 효과크기에 재차 REM 메타분석을 다시 수행하여 summary effect size (sES)와 이의 95% CI를 산출하였다. 메타분석 시행에 있어 이질성 여부 확인은 I-squared 값(%)이 50% 이상일 때 이질성이 있다고 판정하였다. Publication bias 여부를 확인하기 위하여 Funnel plot 과 Egger's test for small-study effects를 시행하였다 [27,28]. 추가로 성별 및 부위별로 하부군 분석 (subgroup analysis)를 수행하였다.

또한 감귤섭취 수준에 따른 Dose-response relationship을 알아보기 위하여 random effects dose-response meta-regression (RE-DRMR)을 시행하였다 [29]. 용량의 결정은 구간 내 중앙값을 반영하였고, 최소 섭취군의 구간이 열린 경우는 하한값을 0으로 삼았다. 만약 최대 섭취군의 구간이 열린 경우는 인접 구간의 중앙값 간격을 적용하였다 [30,31]. 감귤섭취 용량의 단위는 gram/day로 정하였다. 통계적 유의수준을 5%로 정했으며, STATA version 14 (www.stata.com) 통계프로그램을 이용하였다.

Results

Figure 1은 Searching 과정과 Evaluating 과정을 거쳐 분석에 사용할 논문들을 최종적으로 선정한 일련의 과정을 나타낸 흐름도이다. Fang et al. [11] 에서 메타분석의 대상으로 선정한 8개 논문을 중심으로 PubMed 와 Scopus 두 Databases 에 CDT를 적용한 결과 12,164개 목록을 얻어내었다. 여기에 hand searching 과정에서 얻어낸 32개 목록을 합친 후, 8,025개의 중복된 목록을 제거하였다. 중복 제거 후 남은 4,171개 목록에 있어, 초록에 근거하여 3,712개의 목록을 제거하였다. 제거 후 남은 459개 초록은 각 복사본을 확보하여 내용을 평가하면서 454개 논문을 제거하였다. 이에 5개의 코호트 연구 논문을 메타분석 대상논문으로 최종 선정하였다 [16-19, 32]. 총 4,166개 목록을 제거한 기준은 (1) 실험실 연구 310개 (2) 전문가 종설 혹은 체계적 고찰 연구 972개 (3) 기술역학연구 2,430개 (4) 분석역학연구이지만 메타분석에 필요한 정보를 제공하지 않은 연구 398개 (5) 환자-대조군 연구 52개 (6) 위암 사망에 관한 코호트 연구 2개 [14,15] (7) 추적기간을 연장한 코호트 추적 결과가 발표되어 중복된 코호트 연구 2개 [12,13]이었다.

Table 1은 최종 선정된 5개 논문의 코호트의 특성을 정리한 표이다. 지역별로는 미국, 일본, 중국, 네덜란드, 유럽이었다. 추적기간은 최소 4.5, 최고 11년이었으며 감귤섭취수준을 FFQ로 측정하였다. aRR 산출을 위해 5편 모두 흡연을 보정하였으며, 총에너지섭취 보정은 Steevens et al. [17] 을 제외한 나머지 4편에서 이루어졌다. 그러나 Hp 감염정보는 5편

모두 고려하지 않았다.

Figure 2는 각 논문에서 성별 및 부위로 나누어 제시한 aRR과 이의 95% CI에 있어서 ICM법을 적용하여 8 datasets 의 SE와 이의 95% CIs를 구한 다음, REM 메타분석을 수행한 Forest plot 이다. Gonzalez et al. [16]은 부위 뿐만 아니라 전체 대상자에 대한 aRR을 같이 제시하여서 overall gastric cancer 의 RRs 결과를 사용하였다. 총 8 datasets의 sES는 0.87 (95% CI: 0.76-0.99, I-squared = 69.9%)로, 감귤섭취는 위암발생을 억제하며 통계학적으로 유의하였다. moderate heterogeneity 를 보여서 Egger's test를 시행하였는데, small study effects 는 무시할 수 있으며 (coefficient of bias=0.43, p-value=0.89), Funnel plot 에서 좌우대칭성을 확인할 수 있었다 (Figure 3).

위의 해부학적 부위별로 하부군 분석을 하였을 때, non-cardia gastric cancer (NCGC) 는 감귤섭취와 관련성에서 통계적 유의성이 없었으나 (sES=0.95, 95% CI: 0.78-1.15, I-squared=77.5%), cardia gastric cancer (CGC)는 감귤섭취에 발생억제효과 있으며 통계적 유의성을 보였다 (sES= 0.67, 95% CI: 0.55-0.81, I-squared=46.1%) (Table 2). 이에, 5개의 코호트 논문 중 Steevens et al. [17] 논문이 CGC 에 관련한 RE-DRMR 수행이 가능한 정보를 제공하였다. Table 3은 RE-DRMR 의 결과를 제시한 표이다. 통계적 유의성을 보인 가운데 (p-value = 0.002), 하루 100 gram 의 감귤을 섭취할 경우 CGC 발생을 40% 억제하는 효과가 있는 것으로 나왔다 (RR=0.603, 95% CI: 0.439-0.827).

Discussion

이상의 결과들을 정리한다면, 감귤섭취는 위암발생을 13% 감소시키며, 특히 CGC에 있어서 억제 효과가 있다는 것이다. 특히 하부군 분석과 RE-DRMA 분석에서 NGC에 대하여는 통계적 유의성이 없다는 점에서, 감귤 섭취는 CGC의 발생을 억제하는 것으로 추론할 수 있다. antioxidant supplementary 가 항암효과가 없다는 결론이 난 상황에서 [33-35], 본 연구결과는 위암예방의 긍정적인 근거가 된다.

Fang et al. [11]의 SR에 비교해서, 본 연구는 다음 2 가지 방법론에서 구별된다. 첫째, 관련 자료 검색법을 Fang et al. [11]은 전자검색을 한 반면, 본 SR은 CDT를 이용한 검색전략을 사용했다는 점이다 [26]. 같은 가설을 가진 논문들은 기존의 SR에서 선정한 논문들을 인용하거나 유사한 내용을 갖는다는 점에 따라 개발된 검색전략이다. 그 결과, Fang et al. [11] 에서 선정되어야 했을 코호트 연구 하나 [32]를 추가로 확보할 수 있었고, 같은 코호트 자료원 (same cohort source)를 가진 논문을 확인해서 중복한 두 코호트 연구 [12,13]를 제외시킬 수 있었던 것이다. 다시 말해서 CDT를 이용한 adaptive meta-analysis 법은 전자검색식 적용에 비해 누락율을 줄이고 보다 타당한 논문 선정을 할 수 있게 해 주었다 [22,23]. 둘째, 메타분석에 사용하기 위해 선정한 논문에서 추출한 방식이 HLM법이 아니라 ICM 법이라는 점이다 [26]. 그 결과 sES 수준에 있어서 Fang et al. [11] 에 비하여 against null 의 방향을 가졌다 (0.90 versus 0.87). 신뢰구간의 범위는 본 연구에서 더 넓은 신뢰구간

범위 (0.82-1.00 versus 0.76-0.99)를 보였는데, 사망률을 본 두 편의 코호트 연구 [14,15]를 제외하였기에 직접 비교할 수 없다. 그래서 제외한 두 사망률 코호트 연구를 추가한 결과, sES 0.90으로 같은 수준을 보이면서 95% 신뢰구간은 0.83-0.97 으로 더 좁아짐을 확인할 수 있었다 (Table 2). 다시 말해서 ICM법을 적용하여 통계적 검정력을 보다 높여서 더 정확한 결과를 산출할 수 있었다 [26].

본 연구의 한계점은 다음과 같다. 첫째, 선정한 코호트 연구 5편 모두 Hp 감염력에 대한 보정을 하지 않았다는 점이다. 이는 Hp 감염의 중요성이 알려진 것이 최근이라는 점과 함께, 코호트 구축 과정에서 모든 참가자에게 이를 위한 검사를 하기 어렵다는 현실적인 어려움 때문이라고 본다 [10,36]. 그런데 Hp 감염력 정보의 확보와 이를 보정하는 것은 nested case-control studies (NCCS)를 통해 가능하다. 이 NCCS 연구방법을 사용한 12편의 논문에 대한 체계적 고찰연구 [37]에서 Hp 감염은 CGC 발생과는 관련성이 없다고 주장하였다 (sES=0.99, 95% CI: 0.72-1.35). 이 결론을 수용한다면, 본 연구에서 선정한 5편의 코호트 연구가 비록 Hp 감염력을 보정하지 않았다 하더라도 감귤섭취가 CGC 억제효과가 있다는 결론 도출은 타당성을 갖는다고 할 수 있다. 한편 Hansen et al. [38]은 CGC 발생에 있어 Hp infection-induced gastric atrophy 가 위험요인이며, Hp 감염은 직접적인 위험요인이 아니라고 주장하였다. 향후 NCCS 연구설계로 수행한 논문들에 대한 추가적인 SR을 수행할 필요가 있겠다. 둘째, 위암을 부위로 나누어 결론을 도출하기에는 선정된 코호트 논문의 수가 여전히 적다는 점이다. 2007년 4월까지 발표된 논문 중에서는 2편이 선정되었다가 [8],

2015년 12월까지 발표된 논문 중에서는 5편이 선정되었다 [11]. Bae et al. [8]에서 2편에 대한 메타분석에서 감귤섭취와 위암발생간의 통계적 유의성을 확보할 수 없었지만, 본 연구에서는 3편이 추가되면서 통계적 유의성을 확보할 수 있었다. 그러나 5편 중 위암 발생 부위별로 나누었을 때 3편으로 줄어들었다 [16,17,19]. 본 메타분석에서 NGC가 통계적 유의성을 보이지 않는 것에 있어서, 향후 코호트 추적기간을 연장하면서 pooled analysis를 할 필요가 있겠다. 셋째, CGC에 있어서 RE-DRMR을 수행할 수 있는 정보를 제공한 것은 1편에 불과했다. 그렇지만 감귤 하루 섭취량 100 gram 으로 위암 발생을 40% 감소하는 것으로 나왔다. 반면 Vingelience et al. [39]은 동일한 섭취량으로는 위암 사망률을 낮추지 못한다고 보고하였다 (RR=0.95; 95% CI: 0.85-1.05). 향후 다른 코호트 연구 정보가 더해져서 추가적인 연구를 수행할 필요가 있겠다.

이상의 결론들을 요약한다면, 감귤섭취는 위암발생을 억제하며, 특히 CGC 에 있어서 억제 효과가 있다. 이 추론은 현재 NGC 발생이 감소하는 반면 CGC 발생이 증가하는 추세 [20]에서, 향후 위암 예방의 식생활 개선 전략으로 활용할 수 있겠다.

Conflicts of Interest

The authors declare that they have no conflict of interest.

References

1. Torre LA, Bray F, Siegel RL, Ferlay J, Lortet-Tieulent J, Jemal A. Global cancer statistics, 2012. CA Cancer J Clin. 2015;65(2):87-108.

2. Parkin DM. The global health burden of infection-associated cancers in the year 2002. Int J Cancer. 2006;118(12):3030-44.

3. Wroblewski LE, Peek RM Jr, Wilson KT. Helicobacter pylori and gastric cancer: factors that modulate disease risk. Clin Microbiol Rev. 2010;23(4):713-39.

4. Wroblewski LE, Peek RM Jr. Helicobacter pylori in gastric carcinogenesis: mechanisms. Gastroenterol Clin North Am. 2013;42(2):285-98.

5. Guggenheim DE, Shah MA. Gastric cancer epidemiology and risk factors. J Surg Oncol. 2013;107(3):230-6.

6. Nagini S. Carcinoma of the stomach: A review of epidemiology, pathogenesis, molecular genetics and chemoprevention. World J Gastrointest Oncol. 2012;4(7):156-69.

7. Tsugane S, Sasazuki S. Diet and the risk of gastric cancer: review of epidemiological evidence. Gastric Cancer. 2007;10(2):75-83.

8. Bae JM, Lee EJ, Guyatt G. Citrus fruit intake and stomach cancer risk: a quantitative systematic review. Gastric Cancer. 2008;11(1):23-32.

9. Martínez ME, Marshall JR, Giovannucci E. Diet and cancer prevention: the roles of observation and experimentation. Nat Rev Cancer. 2008;8(9):694-703.

10. Musa-Veloso K, Card JW, Wong AW, Cooper DA. Influence of observational study design on the interpretation of cancer risk reduction by carotenoids. Nutr Rev. 2009;67(9):527-45.

11. Fang X, Wei J, He X, An P, Wang H, Jiang L, et al. Landscape of dietary factors associated with risk of gastric cancer: A systematic review and dose-response meta-analysis of prospective cohort studies. Eur J Cancer. 2015;51(18):2820-32.

12. Botterweck AA, van den Brandt PA, Goldbohm RA. A prospective cohort study on vegetable and fruit consumption and stomach cancer risk in The Netherlands. Am J Epidemiol. 1998;148(9):842-53.

13. González CA, Pera G, Agudo A, Bueno-de-Mesquita HB, Ceroti M, Boeing H, et al. Fruit and vegetable intake and the risk of stomach and oesophagus adenocarcinoma in the European Prospective Investigation into Cancer and Nutrition (EPIC-EURGAST). Int J Cancer. 2006;118(10):2559-66.

14. McCullough ML, Robertson AS, Jacobs EJ, Chao A, Calle EE, Thun MJ. A prospective study of diet and stomach cancer mortality in United States men and women. Cancer Epidemiol Biomarkers Prev. 2001;10(11):1201-5.

15. Jansen MC, Bueno-de-Mesquita HB, Räsänen L, Fidanza F, Menotti A, Nissinen A, et al. Consumption of plant foods and stomach cancer mortality in the seven countries study. Is grain consumption a risk factor? Seven Countries Study Research Group. Nutr Cancer. 1999;34(1):49-55.

16. Gonzalez CA, Lujan-Barroso L, Bueno-de-Mesquita HB, Jenab M, Duell EJ, Agudo A, et al. Fruit and vegetable intake and the risk of gastric adenocarcinoma: a reanalysis of the European Prospective Investigation into Cancer and Nutrition (EPIC-EURGAST) study after a longer follow-up. Int J Cancer. 2012;131(12):2910-9.

17. Steevens J, Schouten LJ, Goldbohm RA, van den Brandt PA. Vegetables and fruits consumption and risk of esophageal and gastric cancer subtypes in the Netherlands Cohort Study. Int J Cancer. 2011;129(11):2681-93.

18. Epplein M, Shu XO, Xiang YB, Chow WH, Yang G, Li HL, et al. Fruit and vegetable consumption and risk of distal gastric cancer in the Shanghai Women's and Men's Health studies. Am J Epidemiol. 2010;172(4):397-406.

19. Freedman ND, Subar AF, Hollenbeck AR, Leitzmann MF, Schatzkin A, Abnet CC. Fruit and vegetable intake and gastric cancer risk in a large United States prospective cohort study. Cancer Causes Control. 2008;19(5):459-67.

20. Forman D, Burley VJ. Gastric cancer: global pattern of the disease and an overview of environmental risk factors. Best Pract Res Clin Gastroenterol. 2006;20(4):633-49.

21. Liberati A, Altman DG, Tetzlaff J, Mulrow C, Gøtzsche PC, Ioannidis JP, et al. The PRISMA statement for reporting systematic reviews and meta-analyses of studies that evaluate healthcare interventions: explanation and elaboration. BMJ. 2009;339:b2700.

22. Bae JM, Kim EH. Citation discovery tools for conducting adaptive meta-analyses to update systematic review. J Prev Med Public Health. 2016;49(2):129-33.

23. Bae JM, Kim EH. Human papillomavirus infection and risk of breast cancer: a meta-analysis of case-control studies. Infect Agent Cancer. 2016;11:14.

24. Bae JM, Kim EH. Hormonal Replacement Therapy and the Risk of Lung Cancer in Women: An Adaptive Meta-analysis of Cohort Studies. J Prev Med Public Health. 2015;48(6):280-6.

25. Bae JM. Human papillomavirus 16 infection as a potential risk factor for prostate cancer: an adaptive meta-analysis. Epidemiol Health. 2015;37:e2015005.

26. Bae JM. Comparison of Methods of Extracting Information for Meta-analysis of Observational Studies in Nutritional Epidemiology. Epidemiol Health. 2016;38:e2016003.

27. Egger M, Davey Smith G, Schneider M, Minder C. Bias in meta-analysis detected by a simple, graphical test. BMJ. 1997;315(7109):629-34.

28. Harbord RM, Egger M, Sterne JA. A modified test for small-study effects in meta-analyses of controlled trials with binary endpoints. Stat Med. 2006;25(20):3443-57.

29. Orsini N, Bellocco R, Greenland S. Generalized least squares for trend estimation of summarized dose-respone data. Stata J. 2006;6(1):40-57.

30. Xu X, Yu E, Liu L, Zhang W, Wei X, Gao X, et al. Dietary intake of vitamins A, C, and E and the risk of colorectal adenoma: a meta-analysis of observational studies. Eur J Cancer Prev. 2013;22(6):529-39.

31. Wang X, Ouyang Y, Liu J, Zhu M, Zhao G, Bao W, et al. Fruit and vegetable consumption and mortality from all causes, cardiovascular disease, and cancer: systematic review and dose-response meta-analysis of prospective cohort studies. BMJ. 2014;349:g4490.

32. Li WQ, Kuriyama S, Li Q, Nagai M, Hozawa A, Nishino Y, et al. Citrus consumption and cancer incidence: the Ohsaki cohort study. Int J Cancer. 2010;127(8):1913-22.

33. Bjelakovic G, Nikolova D, Simonetti RG, Gluud C. Antioxidant supplements for prevention of gastrointestinal cancers: a systematic review and meta-analysis. Lancet. 2004;364(9441):1219-28.

34. Huang HY, Caballero B, Chang S, Alberg AJ, Semba RD, Schneyer CR, et al. The efficacy and safety of multivitamin and mineral supplement use to prevent cancer and chronic disease in adults: a systematic review for a National Institutes of Health state-of-the-science conference. Ann Intern Med. 2006;145(5):372-85.

35. Li P, Zhang H, Chen J, Shi Y, Cai J, Yang J, et al. Association between dietary antioxidant vitamins intake/blood level and risk of gastric cancer. Int J Cancer. 2014;135(6):1444-53.

36. Temple NJ. Nutrition and disease: challenges of research design. Nutrition. 2002;18(4):343-7.

37. Helicobacter and Cancer Collaborative Group. Gastric cancer and Helicobacter pylori: a combined analysis of 12 case control studies nested within prospective cohorts. Gut. 2001;49(3):347-53.

38. Hansen S, Vollset SE, Derakhshan MH, Fyfe V, Melby KK, Aase S, et al. Two distinct aetiologies of cardia cancer; evidence from premorbid serological markers of gastric atrophy and Helicobacter pylori status. Gut. 2007;56(7):918-25.

39. Vingelience S, Chan DSM, Aune D, Vieira AR, Polemiti E, Stevens C, et al. An update of the WCRF/AICR systematic literature review on esophageal and gastric cancers and citrus fruits intake. Cancer Causes Control. 2016;27:837-51.

국문요약

항산화제 첨가제가 항암효과가 없다는 결론이 난 상황에서, 감귤섭취가 위암발생을 억제한다는 기존의 체계적 고찰연구에 대한 갱신 메타연구를 수행하였다. PubMed와 Scopus에서 제공하는 citation discovery tools를 활용하여 총 5편의 코호트 연구 논문을 선정하였다. 감귤섭취에 따라 위암발생을 13% 억제하며 (sES=0.87, 95% CI: 0.76-0.99, I-squared: 69.6%), 특히 위문부 위암을 억제한다고 나왔다 (sES= 0.67, 95% CI: 0.55-0.81, I-squared=46.1%), 또한, 하루 100 gram 감귤섭취를 하면 CGC 를 40% 억제하는 것으로 나왔다 (RR=0.60, 95% CI: 0.44-0.83).

Table 1. The selected cohort studies for gastric cancer incidence

First author (year of publication) [reference number]	Cohort population	Size of cohort & Incident Cases (year of follow-up)	Measure of intake (units)	Citrus fruits	Quantity of intake	adjusted RR	95% CI	P value of test for trend	adjusted for total energy intake	adjusted for *Helicobacter pylori* infection
Freedman (2008) [19]	NIH-AAPR Diet and Health Study	490,802 & 394 (4.5)	FFQ (daily serving /1,000K calories)	Oranges, tangerines, tangelos, grapefruits	0.08	1.0	(cardia)	-	Yes	No
					0.46	0.73	0.52-1.03			
					1.12	0.88	0.62-1.23			
					0.08	1.0	(non-cardia)	-		
					0.46	1.15	0.80-1.67			
					1.12	1.36	0.96-1.94			
Li (2010) [32]	Ohsaki Cohort Study	42,470 & 313 (9)	FFQ (times/week)	Citrus	≤ 2	1.0	-	0.626	Yes	No
					3-4	0.95	0.76-1.18			
					daily	0.94	0.74-1.20			
Epplein (2010) [18]	Shanghai Women's and Men's Health Studies	132,311 & 338 (13)	FFQ (gram/day)	Tangerines, organs, grapefruit	≤6.1	1.0	(women)	0.86	Yes	No
					>6.1-17.7	1.00	0.68-1.46			
					>17.7-31.9	1.05	0.71-1.53			
					>31.9	0.94	0.62-1.42			
					≤1.6	1.0	(men)	0.34		
					>1.6-≤6.3	0.84	0.52-1.36			
					>6.3-≤18.0	1.02	0.65-1.62			
					≤18.0	0.70	0.41-1.18			
Steevens (2011) [17]	The Netherlands Cohort Study on diet	4,035 & 616 (16.3)	FFQ (gram/day)	Lemon(juice), grapefruit (juice),	0	1.0	(Cardia)	0.003	No	No
					8	0.76	0.47-1.22			

	and cancer			mandarins, orange (juice)	33	0.54	0.32-0.92			
					77	0.55	0.32-0.94			
					156	0.38	0.21-0.69			
					0	1.0	(Non-cardia)	0.46		
					8	0.86	0.61-1.21			
					33	0.89	0.62-1.27			
					77	0.99	0.70-1.40			
					156	0.80	0.56-1.15			
Gonzalez (2012) [16]	EPIC-EURGAST	477,312 & 683 (11)	FFQ	Citrus	Q1	1.0	(Overall)	0.07	Yes	No
					Q2	0.78	0.62-0.99			
					Q3	0.84	0.67-1.07			
					Q4	0.63	0.49-0.82			
					Q5	0.87	0.68-1.12			
					Q1	1.0	(Cardia)	0.01		
					Q2	0.73	0.49-1.08			
					Q3	0.73	0.48-1.11			
					Q4	0.54	0.34-0.85			
					Q5	0.61	0.38-1.00			
					Q1	1.0	(Non-cardia)	0.46		
					Q2	0.93	0.64-1.34			
					Q3	1.07	0.75-1.53			
					Q4	0.79	0.54-1.16			
					Q5	1.25	0.86-1.80			

CI (confidence intervals) FFQ (food frequency questionnaire) RR (relative risk)

Table 2. Subgroup analysis by anatomical location of stomach cancer

Subgroup	Reference number	sES	95% CI	I-squared (%)
Incidence	16-19, 32	0.87	0.76, 0.99	61.3
Non-cardia	16, 17, 19	0.95	0.78, 1.15	77.5
Cardia	16, 17, 19	0.67	0.55, 0.81	46.1

CI (confidence intervals) sES (summary effective size)

Table 3. Relative risks (RR)[1] of dose-response meta-regression using relative risks of cardia gastric cancer in Steevens et al. [17]

Intake of citrus fruits	RR	95% confidence interval
1	0.995	0.992, 0.998
10	0.951	0.921, 0.981
20	0.904	0.848, 0.963
25	0.881	0.814, 0.924
50	0.776	0.663, 0.910
75	0.684	0.540, 0.868
100	0.603	0.439, 0.827

[1]P-value = 0.002

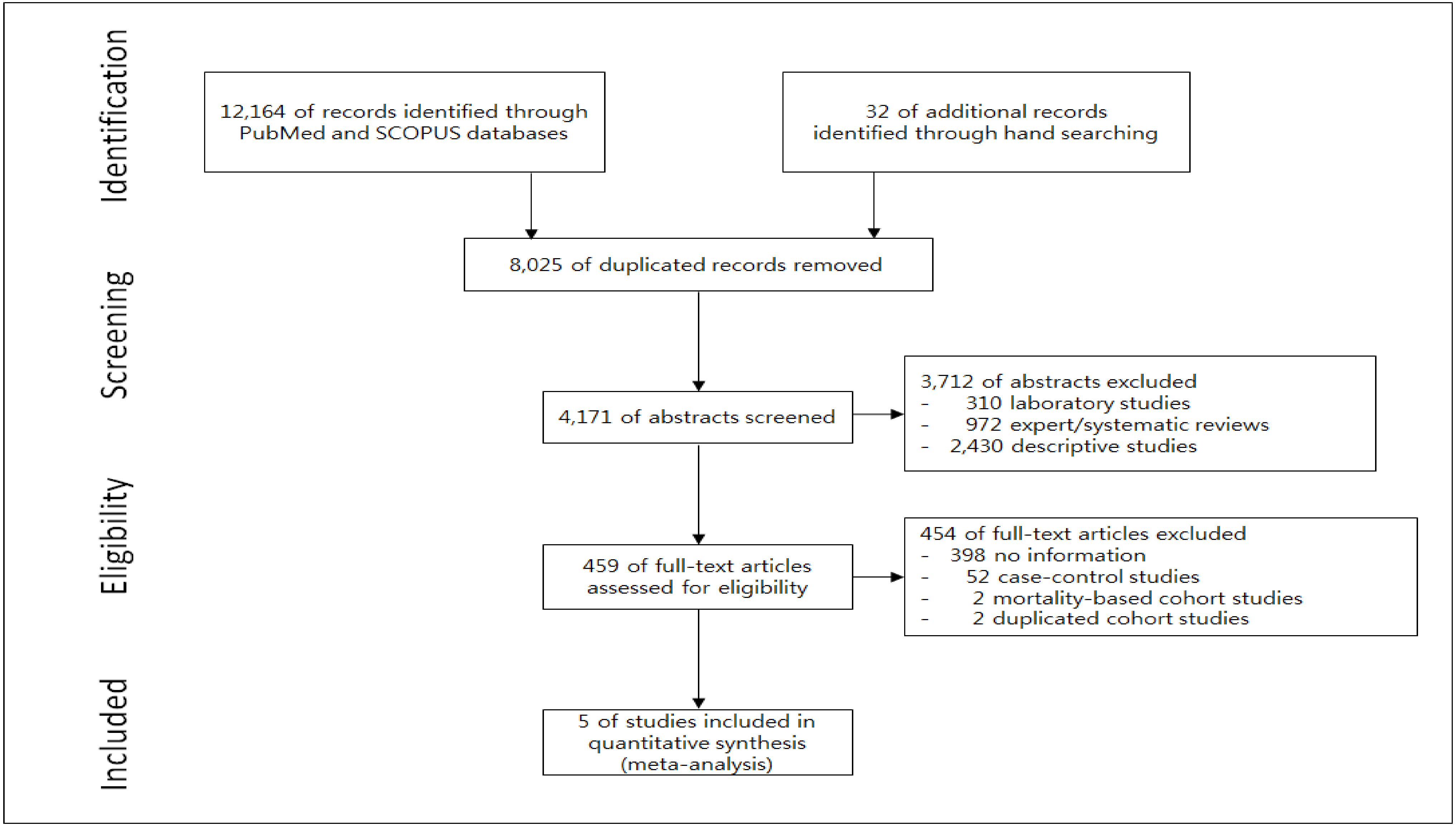

Figure 1. Flow chart of article selection

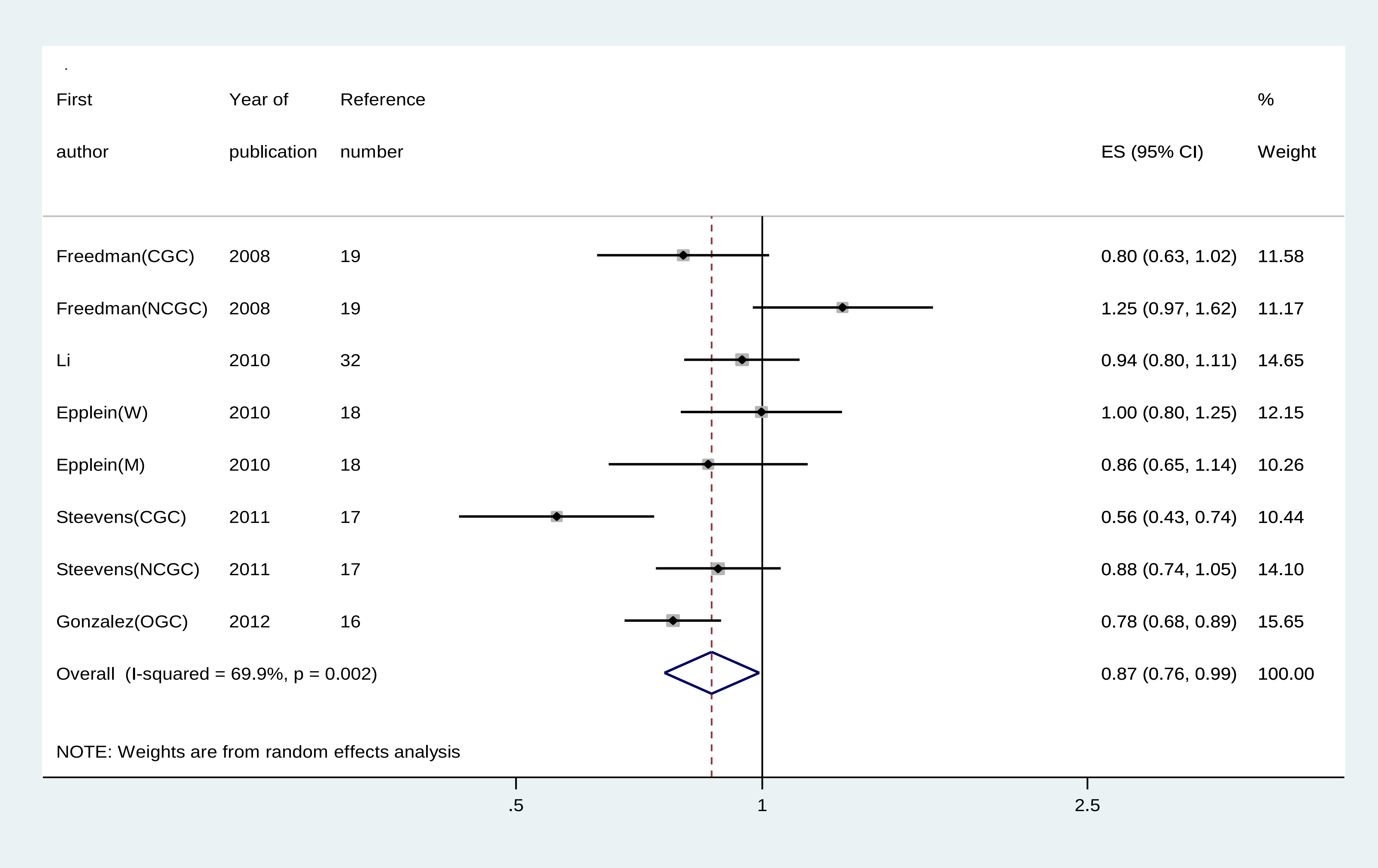

Figure 2. The forest plot of effect size (ES) and its 95 % confidence intervals (CI) using a random effect model in 8 datasets from 5 cohort studies

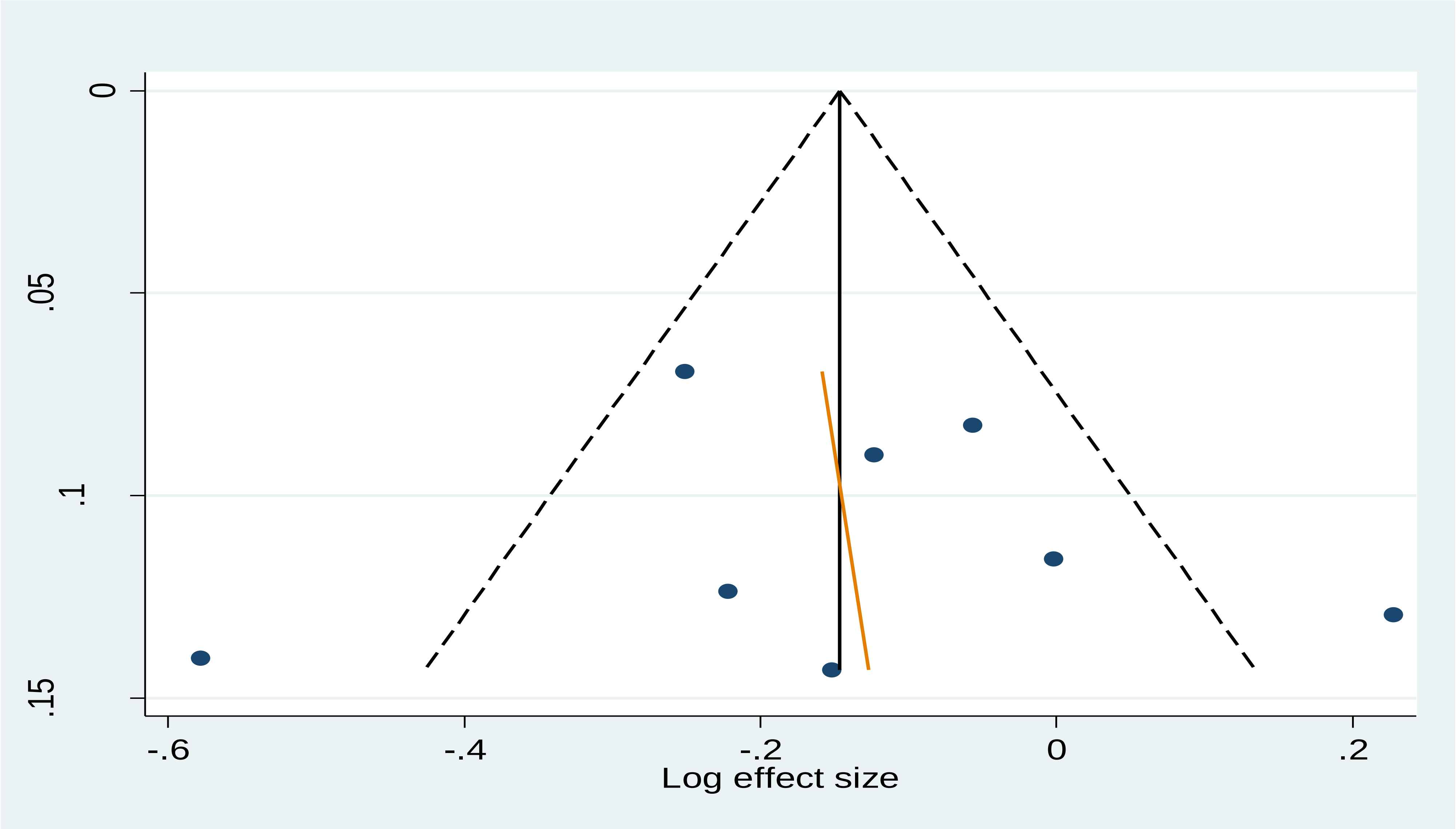

Figure 3. Funnel plot with pseudo 95% confidence limits in 8 datasets from 5 cohort studies
